# Enhanced Light-Harvesting Efficiency and Adaptation: A Review on Visible-Light-Driven Micro/Nanomotors

**DOI:** 10.34133/2020/6821595

**Published:** 2020-08-25

**Authors:** Dekai Zhou, Rencheng Zhuang, Xiaocong Chang, Longqiu Li

**Affiliations:** ^1^Key Laboratory of Microsystems and Microstructures Manufacturing, Harbin Institute of Technology, Harbin, Heilongjiang 150001, China; ^2^State Key Laboratory of Robotics and System, Harbin Institute of Technology, Harbin, Heilongjiang 150001, China

## Abstract

As visible light accounts for a larger proportion of solar energy and is harmless to living organisms, it has the potential to be the energy source of micro/nanomotors, which transform visible-light energy into mechanical motion, for different applications, especially in environmental remediation. However, how to precisely control the motion of visible-light-driven micro/nanomotors (VLD-MNMs) and efficiently utilize the weak visible-light photon energy to acquire rapid motion are significant challenges. This review summarizes the most critical aspects, involving photoactive materials, propulsion mechanisms, control methods, and applications of VLD-MNMs, and discusses strategies to systematically enhance the energy-harvesting efficiency and adaptation. At first, the photoactive materials have been divided into inorganic and organic photoactive materials and comprehensively discussed. Then, different propulsion mechanisms of the current VLD-MNMs are presented to explain the improvement in the actuation force, speed, and environmental adaptability. In addition, considering the characteristics of easy control of VLD-MNMs, we summarized the direction, speed, and cluster control methods of VLD-MNMs for different application requirements. Subsequently, the potential applications of VLD-MNMs, e.g., in environmental remediation, micropumps, cargo delivery, and sensing in microscale, are presented. Finally, discussions and suggestions for future directions to enhance the energy-harvesting efficiency and adaptation of VLD-MNMs are provided.

## 1. Introduction

Micro/nanomotors, which are typical devices or systems with micro/nanoscale dimensions, show significant characteristics such as small size, easy propulsion, and controllability. Considerable artificial micro/nanomotors have been designed, fabricated, and studied in the last decade. Difficult challenges in the application of micro/nanomotors in various fields, ranging from biomedicine and sensing to environmental remediation, have been overcome in different environments [1–9]. To enhance the drive performance, controllability, and environmental suitability of micro/nanomotors, different types of input energies, e.g., chemical, magnetic, light, thermal, electric, and acoustic energies, were used to drive and control their motion. To date, different propulsion mechanisms have been used in micro/nanomotor: (1) self-electrophoresis [10] and self-diffusiophoresis [11] in chemically powered motors, (2) bubble propulsion [12, 13], (3) thermophoresis [14], (4) magnetic field propulsion [15, 16], (5) electrophoresis [17], (6) light-driven propulsion [18, 19], and (7) acoustophoresis [20, 21]. Among all abovementioned input energies, light energy is clean and has unique advantages. First, light is easy and inexpensive to achieve, which is of significant potential in environmental treatment. Second, light can be wirelessly transmitted and remotely controlled, which shows substantial advantages for the indirect control of micro/nanomotors. Finally, for all driving methods, the overall system of light-driven micro/nanomotors is simple, which is suitable for use over a wide range. Due to these outstanding features, light-driven micro/nanomotors have become one of the most attractive research topics. Initially, researchers discovered the photoisomerization phenomenon of photochromic materials, and several excellent light-driven molecular machines were developed [22–27]. In 2006, Kline and Sen found that silver disks can pump or attract particles in a hydrogen peroxide solution under UV light [28]. Subsequently, solid-state micro/nanomotors gradually became another hot topic in the field of light-driven micro/nanomotors owing to their fast speed, strong impetus, and wide range of motion.

**Figure 1:**
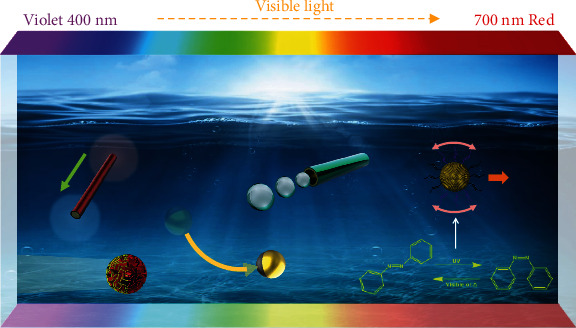
Schematic of different types of visible-light-driven micro/nanomotors. Reprinted with permission from the American Chemical Society [29, 30].

Over the past few years, the design, fabrication, and application of both UV-driven [31, 32] and NIR-driven [33, 34] micro/nanomotors have been reviewed. However, as the proportion of UV and NIR light is low in sunlight and UV light is harmful to human beings, a huge gap exists between scientific discoveries and applications for light-driven micro/nanomotors. Therefore, recently, significant attention has been paid to visible-light-driven micro/nanomotors (VLD-MNMs) as shown in Figure 1 due to the unique advantages of visible light. At first, visible light accounts for a larger proportion (43%) of the solar energy than UV and NIR light; it is a theoretically unlimited fuel source in nature [35]. Thus, VLD-MNMs own huge application potential in environmental remediation, especially in large-scale wastewater treatment as sunlight can be utilized as the energy source. Second, the photon energy of visible light falls between UV and NIR light which makes it own the advantages of both. On the one hand, it is similar to NIR light which is harmless to human beings compared with UV light. Simultaneously, it is the same with UV light which can trigger more driven modes for micro/nanomotors, e.g., photoelectric effect, photochemical reaction, and photochromic reaction, with weaker light intensity than NIR-light-driven micro/nanomotors by photothermal effect. Recently, Dong et al., Wang et al., and Zhou et al. reported a series of VLD-MNMs including BiOI/metal [29], Cu_2+1_O [36], Si/Au [37], Cu_2_O/Au [38], and iron oxide/Au [39] micro/nanomotors. Dai et al., Chen et al., and Aubret et al. concentrated more on the control methods of VLD-MNMs [40–42]. Villa et al. and Moo et al. demonstrated that VLD-MNMs are capable of achieving specific functions [43, 44]. Pollard et al., Balzani et al., and Li et al. reported a series of visible-light-driven molecular machines such as rotary motors [45, 46], molecular shuttles [47–49], and molecular switches [50]. A summary of the representative examples of VLD-MNMs is presented in Table 1. In the near future, VLD-MNMs will become another research direction for light-driven micro/nanomotors and will have a profound impact on the development of micro/nanomachines.

In fact, it is essential to select appropriate materials to improve the absorption ability of VLD-MNMs. In addition, efficient control of the motion of VLD-MNMs and utilization of the weak visible-light photon energy to acquire rapid movement are significant challenges. It is important to systematically understand all the basic requirements before fabricating VLD-MNMs for application in different areas. However, to the best of our knowledge, to date, no work has presented a comprehensive overview of the materials, propulsion mechanisms, control methods, and applications of VLD-MNMs. Hence, the objective of this work is to fill this knowledge gap by providing an overview of VLD-MNMs, which will be helpful for researchers who want to conduct research on VLD-MNMs.

In this review, after a brief introduction of micro/nanomotors, we have discussed the advantages of VLD-MNMs. In Section 2, photoactive materials, including photocatalytic materials, photosensitive materials, photothermal materials, photochromic materials, and dyes, for the design of VLD-MNMs are illustrated. Then, the propulsion mechanisms involving self-electrophoresis, self-diffusiophoresis, bubble propulsion, and photochromic reaction propulsion of VLD-MNMs are introduced. Subsequently, we have summarized the direction, speed, and cluster control methods of VLD-MNMs. Additionally, typical applications of VLD-MNMs in water treatment, micropumps, cargo delivery, and sensing in microscale are presented. Finally, helpful discussions and research opinions are provided. We hope that this review will provide useful guidance for researchers, especially to enhance the propulsion and absorption efficiency of VLD-MNMs, and promote the practical applications of VLD-MNMs in the future.

## 2. Photoactive Materials

Photoactive materials are the core part of the material system of VLD-MNMs as they absorb and convert visible-light energy into other forms of energy, including electrical, chemical, and thermal energies, for the movement of micro/nanomotors [71]. The photoactive materials used for micro/nanomotors can be classified as inorganic photoactive materials and organic photoactive materials. Based on the energy generated by conversion, the inorganic photoactive materials can be divided into photocatalytic, photosensitive, and photothermal materials. The organic photoactive materials mainly include organic photochromic materials and dyes, as illustrated in Table 2. Basically, the performance of VLD-MNMs is highly dependent on the materials used for their synthesis. Different materials are used corresponding to the propulsion mechanism and the control method. Hereinafter, we have briefly discussed the photoactive materials used to synthesize VLD-MNMs.

### 2.1. Inorganic Photoactive Materials

The energy of absorbed visible light can be transformed into different forms by different inorganic photoactive materials. Basically, some inorganic materials can absorb photon energy and convert it into electric energy to catalyze solution decomposition based on the photoelectric effect [72]. On the contrary, some inorganic salts photolyze into ions under light irradiation [73]. Furthermore, some inorganic materials exhibit a photothermal effect, and photon energy is converted into heat when these materials are irradiated by visible light [74]. Based on the aforementioned principles, the inorganic photoactive materials can be classified into three categories: photocatalytic, photosensitive, and photothermal materials.

#### 2.1.1. Photocatalytic Materials

**Figure 2:**
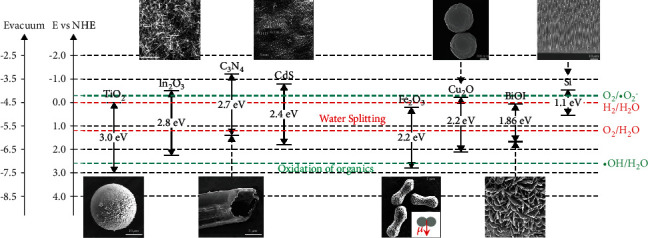
Energy bands of materials used in VLD-MNMs [81, 82]. From left to right: SEM images of TiO_2_/Au [83], In_2_O_3_ [84], C_3_N_4_ [77], CdS/Ta_2_O_5_ [85], Fe_2_O_3_ [86], Cu_2_O/Au [38], BiOI [29], and Si [53] micro/nanomotors. Reprinted with permission from the American Chemical Society, Elsevier, John Wiley & Sons, Inc., and Royal Society of Chemistry.

The photocatalytic materials used for synthesizing VLD-MNMs are mainly semiconductor materials that usually have two energy bands, namely, the valence band (VB) and the conduction band (CB). The region between the VB and CB is called band gap (*E*_*g*_) [73]. These materials perform two tasks in the VLD-MNM system: light absorption and catalytic deposition. Briefly, when photon energy (*hν*) is higher than *E*_*g*_, the inner electrons/holes are excited to catalyze solution decomposition. This photocatalytic process will form a concentration gradient to propel the micro/nanomotors. Under ideal conditions, the photocatalytic materials used for synthesizing VLD-MNMs should satisfy the following conditions: (1) their band gap should be less than the energy of visible light and they should show a high absorption efficiency for visible light; (2) their CBs and VBs should match the reaction potential of the solution; (3) they should be chemically stable under visible light; and (4) they should have a high catalytic efficiency. Considering the frequency of visible light, we can calculate the energy of visible light based on the Planck Einstein equation:

(1) $$\begin{array}{c} E=\frac{hc}{\lambda }, \end{array}$$

where *h* denotes the Planck constant, *c* is the light velocity, and *λ* denotes the wavelength of light. Visible-light-active photocatalysts usually have a band gap between 1.5 and 3.1 eV.  Table 3 presents semiconductor materials as well as their *E*_*g*_, which can absorb visible light and drive the micro/nanomotors to move. Their energy bands are shown in Figure 2, which may be useful for further selection of core materials for VLD-MNMs.

As most of the semiconductor materials have poor light absorption ability and low catalytic activity, it is difficult to find any existing semiconductor material that can perfectly meet the requirements of VLD-MNMs. Therefore, researchers have tried different methods to improve the absorption ability and photocatalytic efficiency of VLD-MNM materials.

The visible-light absorption ability can be improved by the following three methods [87, 88]. (1) Changing the crystal structure: the changes in the crystal structure mainly include the improvement of crystallinity and the formation of crystalline phases. These changes will affect the number of defects and the band gap of materials, which would eventually alter the light absorption characteristics. For instance, when TiO_2_ is annealed, its crystalline phase is converted to B-TiO_2_, which has visible-light absorption ability [51]. During annealing, the crystal structure of TiO_2_ transforms from the amorphous state to the crystalline anatase phase of titania and oxygen vacancies are created in B-TiO_2_, which cause a change in the absorption characteristics. (2) Changing the surface state: surface states, such as roughness, porosity, and area, affect the contact between the materials and the solution, which in turn affects the absorption of light. Pourrahimi et al. investigated the influence of the surface profile of materials on the ZnO/Pt-based Janus micromotors [69]. When the ZnO particles react with the H_2_O_2_ solution, surface ZnO is oxidized to ZnO_2_ [89]. This change will influence the band gap of ZnO and improve the visible-light absorption ability. Similar work was also reported by Xu et al. [85]. (3) Changing the morphology: in general, there are four major types of morphologies of VLD-MNMs including microsphere, nanotubular, nanorod, and asymmetric branches, which have little impact on light absorption efficiency. In order to improve the light absorption efficiency, some special morphologies such as nanocaps are needed as these unique structured Au can be activated by visible light based on the surface plasmon effect. Considering this, a nanocap-shaped Au/TiO_2_ nanomotor was investigated by Wang et al., showing its motion can be increased by visible-light irradiation [52], while the Au/TiO_2_ micromotors of the microsphere type can only be propelled by UV light as reported [83]. The morphology of these micro/nanomotors has a substantial impact on their absorption ability which affects the motion of micro/nanomotors.

Doping is an effective method to improve the photocatalytic efficiency of semiconductor materials as it can effectively enhance the electron transport efficiency and reduce the combination of electrons and holes. There are two implementations of doping: inside and on the surface of the materials. For the case of doping inside the materials, Wang et al. chose nitrogen-doped carbon nanotubes (N-CNTs) to compensate for the deficiencies of Cu_2_O [58]. They built highways for activated electrons by adding N-CNTs, which could reduce the recombination of electrons/holes. For the case of doping on the surface of materials, Wang et al. and Zheng et al. reported a series of micro/nanomotors doped with Pt nanoparticles, which transferred the catalytic location from the semiconductor to Pt [53, 55]. It was found that the Pt nanoparticles can improve the catalytic efficiency of semiconductor materials by enhancing electron transfer.

#### 2.1.2. Photosensitive Materials

Photosensitive materials are unstable and easily decompose under light irradiation. Photosensitive materials are mainly inorganic salts, especially silver ion salts (AgBr, AgCl, or AgI) [73]. Under ultraviolet-light or strong visible-light irradiation [63], silver halides are decomposed and reacted with the solution. For example, in an aqueous solution containing silver ions, the silver ions in the solution will be reduced to silver and water will be oxidized to oxygen, which may undergo further oxidation reactions. Eventually, the solution will contain O_2_, Ag, X^−^, and H^+^ and generate an ion concentration gradient to drive the VLD-MNMs.

#### 2.1.3. Photothermal Materials

The inorganic photoactive materials capable of converting light energy into thermal energy are called photothermal materials; these materials generate photothermal effect under light irradiation and form a temperature gradient in the solution. Photothermal materials generally include carbon materials and metallic materials. Note that metallic materials also generate localized surface plasmon resonance, which can strongly enhance the photothermal effect [90]. Under light irradiation, mobile carriers will be generated in gold nanocrystals and converted into hot electrons through electron-electron collisions. The thermal energy of the hot electrons is released into the solution around the nanocrystals; this causes a change in the temperature of the solution and hence generates a temperature gradient. Photothermal materials usually show excellent absorption characteristics only under near-infrared light [91–93]. Therefore, these materials are rarely used in VLD-MNMs.

### 2.2. Organic Photoactive Materials

Organic photoactive materials include organic photochromic materials and dyes. The organic photochromic materials absorb light energy through photochromic reactions that induce changes in their physical and chemical properties. Dyes can convert light energy into electrical energy and inject electrons into semiconductor materials. This can widen the light absorption range and therefore improve the utilization efficiency of light energy.

#### 2.2.1. Photochromic Materials

Under light irradiation, the organic photochromic materials will undergo isomer conversion, which would result in physical and chemical changes [94]. By utilizing these physical and chemical changes, some VLD-MNMs have been designed, which present unique motion characteristics.

**Figure 3:**
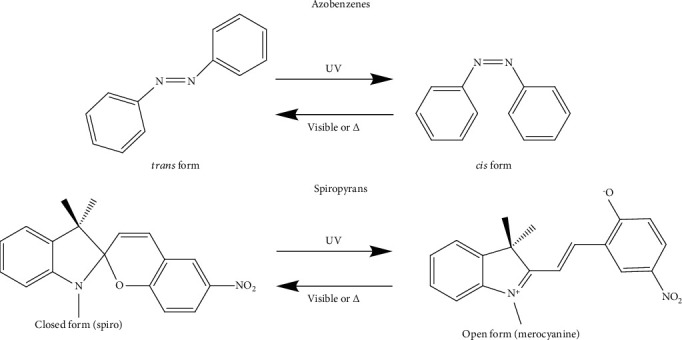
The photochromic materials azobenzenes and spiropyrans used in VLD-MNMs.

Azobenzenes and spiropyrans are two typical organic photochromic materials commonly used in VLD-MNMs, as shown in Figure 3. Under light irradiation, the planar *trans*-isomer of azobenzenes can undergo conversion to the bent *cis*-isomer through the isomerization of the -N=N- bond [95]. Similar to photochromic reactions, this transformation process is accompanied by changes in the physical and chemical properties. These changes can provide guidance for the design of VLD-MNMs using azobenzenes [96, 97]. Spiropyrans are another type of photochromic materials commonly used in the synthesis of VLD-MNMs. During photoisomerization, their stable/metastable states can be switched due to the changes in surface free energy induced by UV and visible light [98].

#### 2.2.2. Dyes

Inspired by their application in the synthesis of dye-sensitized solar cells, dyes, as organic photoactive materials, have been applied in VLD-MNMs [99–101]. When exposed to visible light, dyes absorb photon energy, and the electrons in the ground state are excited, forming photogenerated electrons. The photogenerated electrons will move to the semiconductor and will be converted into electrical energy, while holes will remain in the dye molecules. By loading dyes into semiconductor materials, the light utilization ability of semiconductor materials can be significantly enhanced. Furthermore, Zheng et al. investigated a coded visible-light-driven micromotor by loading different dyes (N719, D5, and SQ2) on it [102].

It was found that inorganic photocatalytic materials play a dominant role in the photoactive system of VLD-MNMs. VLD-MNMs based on inorganic materials, e.g. Cu_2_O, Fe_2_O_3_, and TiO_2_, show significant potential for water treatment because of their easy preparation, low cost, and surface functionalization properties. In contrast, organic VLD-MNMs can be used in precision areas, such as molecular machines, flexible microstructures, and biomedical engineering, due to their small size and flexibility. To develop photoactive materials for VLD-MNMs, it is important to improve the visible-light absorption performance and catalytic ability of all the existing photoactive materials. The development of photoactive materials that can absorb the full spectrum of sunlight and catalyze the decomposition of solutions, including water and organic solvents, is a major research direction in the future. Meantime, the development of microscale, removable, and various medical machinery, for example, microscalpel, microsyringe, and microbandage, which can achieve motion, complex shape, and function deformation of VLD-MNMs, will significantly expand the application of VLD-MNMs by combining the advantages of both inorganic and organic materials.

## 3. Propulsion Mechanisms

As is well known, the Reynolds number of micro/nanomotors moving in a fluid is quite low. Thus, a strong driving force is required to push the micro/nanomotors to overcome the viscous force when they move in a viscous fluid. Therefore, investigating the driving mechanism is generally the main objective of all relevant studies. VLD-MNMs are usually achieved by breaking the symmetry of the system. When photons hit the surface of VLD-MNMs, an electrolyte concentration gradient, a nonelectrolyte concentration gradient, and bubbles are generated in the surrounding solution, thereby pushing the micro/nanomotors forward. Based on the materials used in VLD-MNMs, the propulsion mechanisms can be divided into self-electrophoresis, self-diffusiophoresis, bubble propulsion, and photochromic reaction propulsion.

### 3.1. Self-Electrophoresis

Self-electrophoresis-based VLD-MNMs always have an asymmetric Janus structure. Basically, when the VLD-MNMs are irradiated, the solvent is decomposed, and a reduction/oxidation reaction occurs at different ends of the VLD-MNMs due to their asymmetric structure. Then, the charged ions will generate a local electric field resulting from the chemical gradient and drive the micro/nanomotors. Therefore, the main mechanism of self-electrophoresis-based VLD-MNMs involves the generation of a local electric field of ions. The asymmetric distribution of charged ions relies on the asymmetric structure or asymmetric light exposure.

The velocity of the motors propelled by self-electrophoresis can be described as follows [103]:

(2) $$\begin{array}{c} U=\frac{\zeta \varepsilon }{\mu }E, \end{array}$$

where *ζ* denotes the zeta potential of the micromotor surface, *ε* denotes the permittivity of the solution, *μ* is the fluid viscosity, and *E* is the electric field intensity.

**Figure 4:**
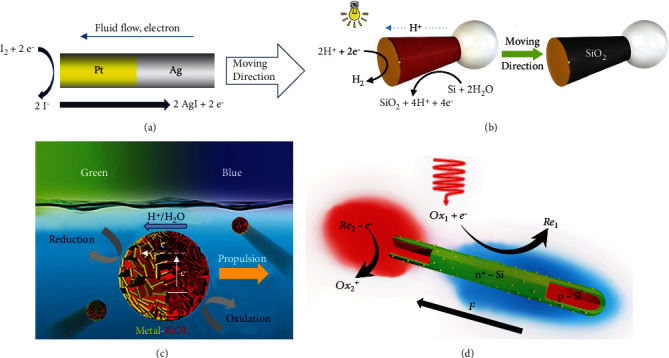
Visible-light-driven self-electrophoresis-propelled micro/nanomotors. Metal/metal micro/nanomotors: (a) silver/Pt nanomotors [57] (reprinted with permission from the American Chemical Society). Semiconductor/metal micro/nanomotors: (b) Si/Au micromotors [37] (reprinted with permission from the Royal Society of Chemistry) and (c) BiOI/metal Janus micromotors [29] (reprinted with permission from the American Chemical Society). *n*-type semiconductor/*p*-type semiconductor micro/nanomotors: (d) schematic of the visible-light-driven silicon nanowires [53] (reprinted with permission from John Wiley & Sons, Inc.).

Generally, two different materials are used to obtain the Janus structure of micro/nanomotors. Based on the different materials used for synthesizing micro/nanomotors, the visible-light-driven self-electrophoresis-propelled micro/nanomotors are mainly classified into three categories: metal/metal micro/nanomotors, semiconductor/metal micro/nanomotors, and *n*-type semiconductor/*p*-type semiconductor micro/nanomotors. (1) Metal/metal micro/nanomotors: this type of nanomotors are generally composed of bimetallic Ag-Pt [57], in which Ag reacts with the iodine solvent and an ion concentration gradient is induced between Ag and Pt sides upon light irradiation. Then, an electroosmotic flow is induced, pushing the nanomotor toward the Ag side, as illustrated in Figure 4(a). (2) Semiconductor/metal micro/nanomotors: Zhou et al. proposed a tadpole-shaped Si-Au micromotor which is a typical semiconductor-metallic structure of self-electrophoresis-propelled micromotors. The Si-Au micromotor can absorb visible light and catalyze the decomposition of deionized water or organic solvents, forming an electric field to drive its motion [37], as shown in Figure 4(b). Similar work was also performed by Dong et al. [29], as shown in Figure 4(c). (3) *n*-type semiconductor/*p*-type semiconductor micro/nanomotors: Wang et al. proposed a type of visible-light-driven nanomotors consisting of an *n*-type semiconductor and a *p*-type semiconductor. These nanomotors have a core-shell structure in which the inner *p*-type silicon core is coated with *n*-type silicon shell [53], as illustrated in Figure 4(d). Activated electrons and holes are transferred to specific semiconductors, and a photovoltage is generated between the *p*-type and *n*-type silicon under visible-light irradiation; thus, chemical reactions occur on different surfaces.

### 3.2. Self-Diffusiophoresis

Similar to the aforementioned self-electrophoresis propulsion mechanism, self-diffusiophoresis is another propulsion mechanism for VLD-MNMs. Owing to the asymmetric structures, such as Janus particles, or asymmetric photo exposure of micro/nanomotors, the photocatalytic reactions occur asymmetrically and generate ion or molecular concentration gradients. Therefore, the concentration gradient can create a fluid flow pushing the micro/nanomotors forward. According to the different reactants produced, self-diffusiophoresis can be classified into electrolyte diffusiophoresis and nonelectrolyte diffusiophoresis.

#### 3.2.1. Electrolyte Diffusiophoresis

Electrolyte diffusiophoresis is a type of diffusiophoretic flow that is mainly governed by the electrophoretic effect. The velocity of motors based on self-diffusiophoresis can be described by the following equation [103]:

(3) $$\begin{array}{c} U=\frac{\nabla c}{c_{0}}\left[\left(\frac{D^{+}-D^{-}}{D^{+}+D^{-}}\right)\left(\frac{k_{B}T}{e}\right)\frac{\varepsilon \left(\zeta_{p}-\zeta_{s}\right)}{\eta }\right]+\frac{\nabla c}{c_{0}}\left\{\left(\frac{2\varepsilon k_{B}^{2}T^{2}}{\eta e^{2}}\right)\right.\times \left.\left[\ln\left(1-{\left(\tan\frac{e\zeta_{s}}{4k_{B}6T}\right)}^{2}\right)-\ln\left(1-{\left(\tan\frac{e\zeta_{p}}{4k_{B}T}\right)}^{2}\right)\right]\right\}, \end{array}$$

where *c*_0_ is the bulk concentration of ions, ∇*c* denotes the concentration gradient, *D*^+^ and *D*^−^ are the diffusivities of the photoinduced cations and anions, *k*_*B*_ is the Boltzmann constant, *T* is the temperature of the solution, *e* is the charge of the electron, *ε* denotes the dielectric permittivity of the solution, *η* is the fluid viscosity, and *ζ*_*p*_ and *ζ*_*s*_ are the zeta potentials of the particle and substrate, respectively [104].

**Figure 5:**
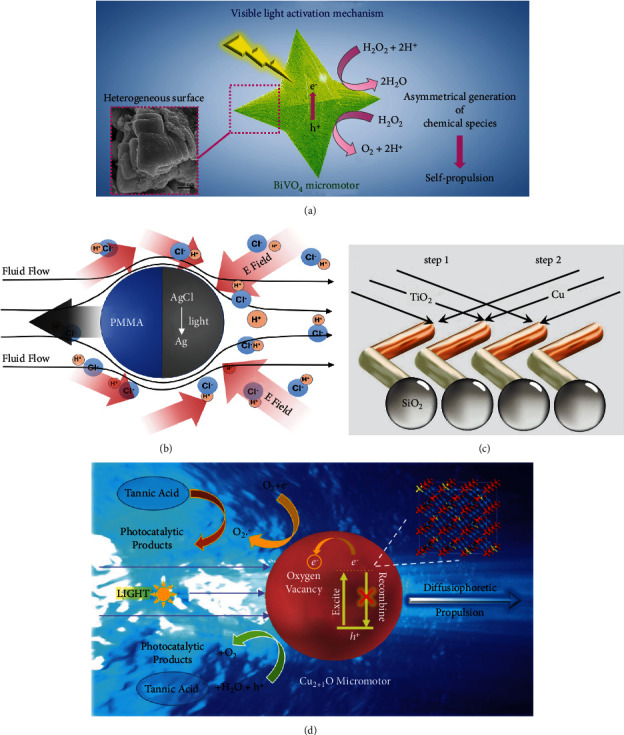
Visible-light-driven self-diffusiophoresis-propelled micro/nanomotors. Electrolyte diffusiophoresis: (a) BiVO_4_ micromotors [43] (reprinted with permission from the American Chemical Society) and (b) PMMA-AgCl micromotors [63] (reprinted with permission from the American Chemical Society). Nonelectrolyte diffusiophoresis: (c) SiO_2_-TiO_2_-Cu_2_O micromotors [61] (reprinted with permission from John Wiley & Sons, Inc.) and (d) Cu_2+1_O micromotors [36] (reprinted with permission from the Royal Society of Chemistry).

Villa et al. reported single-component BiVO_4_ micromotors with star-shaped microstructures that can move based on self-diffusiophoresis propulsion [43], as shown in Figure 5(a). When the BiVO_4_ micromotor is illuminated by visible light, the H_2_O_2_ solution decomposes on its surface and generates superoxide and protons. Due to the discrepancy in the diffusion rates of ions, an electrical field and chemical pressure are created around the motor that push the BiVO_4_ micromotors forward. Another similar example of electrolyte diffusiophoresis was reported by Zhou et al. [63], as illustrated in Figure 5(b).

#### 3.2.2. Nonelectrolyte Diffusiophoresis

In addition to electrolyte diffusiophoresis, nonelectrolyte diffusiophoresis is very common in the self-diffusiophoresis system; however, the forces generated are lower than those resulting from electrolyte diffusiophoresis. Theoretically, the force in the case of nonelectrolyte diffusiophoresis is generated by a pressure gradient resulting from the interaction of solutes with the particle surface [105]. The asymmetric structure or asymmetric irradiation breaks down the symmetry of the pressure distribution and leads to the motion of micro/nanomotors. The velocity of the motors can be calculated as follows [41]:

(4) $$\begin{array}{c} U=\frac{kT}{\eta }KL\nabla C, \end{array}$$

where *k* denotes the Boltzmann constant, *η* denotes the fluid viscosity, *T* is the temperature of the solution, and *K* and *L* are the Gibbs absorption strength and the length of the motor, respectively, and ∇*C* denotes the concentration gradient.

According to the nonelectrolyte diffusiophoresis, a hybrid photoactivated microswimmer is reported, which is driven by a molecular concentration gradient at the catalytic site [61], as shown in Figure 5(c). Meanwhile, by adding tannic acid to the solution as a fuel, Wang et al. found that the Cu_2+1_O microparticles can move under visible-light irradiation. The motion of the Cu_2+1_O microparticles is driven by the concentration gradient of photocatalytic products produced by the decomposition of tannic acid [36], as shown in Figure 5(d).

### 3.3. Bubble Propulsion

Bubble propulsion is widely used for micro/nanomotors. Unlike the case of previous bubble-driven micro/nanomotors, photocatalytic materials (e.g., TiO_2_ or WO_3_) are added to the micro/nanomotors instead of traditional catalytic materials. Under visible-light irradiation, bubbles start to generate and release from one side due to the asymmetric structure of the micro/nanomotors and the asymmetric discharge of bubbles. As bubbles are released from one side of the micro/nanomotors, a driving force is created to push them away from the micro/nanomotors. The velocity of the bubble propulsion motors can be written as follows [106]

(5) $$\begin{array}{c} U\propto N\frac{R_{g}T\rho v_{0}}{\mu aP}\gamma^{2}\frac{k\alpha c}{1+\alpha c}, \end{array}$$

where *T* is the temperature, *N* denotes the number of bubbles, *R*_*g*_ denotes the universal gas constant, *ρ* is the density of O_2_, *v*_0_ is the initial horizontal speed of the detached bubble, *μ* is the viscosity of the fluid, *a* is the radius of the particle, *P* is the ideal gas pressure, *γ* is the surface tension, *k* is the catalytic reaction rate constant, *α* is the Langmuir adsorption constant, and *c* is the bulk concentration of hydrogen peroxide. Meanwhile, owing to the easy-to-control characteristics of visible light, the bubble-propelled VLD-MNMs can flexibly achieve rapid on/off or fast/low behavior.

**Figure 6:**
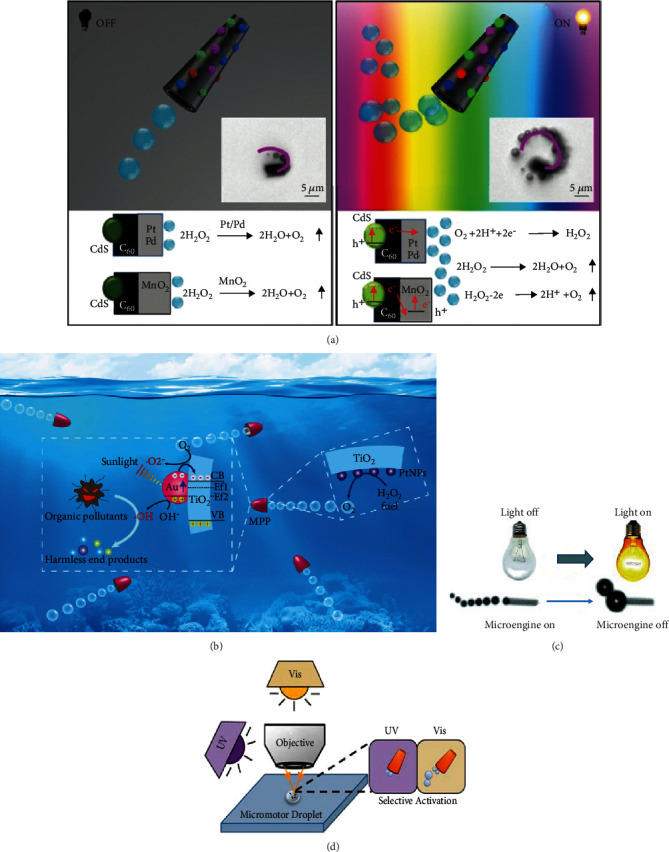
Visible-light-driven bubble-propelled micro/nanomotors: (a) CdS/C_60_ micromotors [67] (reprinted with permission from John Wiley & Sons, Inc.) and (b) TiO_2_/Au micromotors [68] (reprinted with permission from the Royal Society of Chemistry). Visible-light-controlled bubble-propelled micro/nanomotors: (c) Ti/Cr/Pt micromotors [70] (reprinted with permission from John Wiley & Sons, Inc.) and (d) spiropyrans/Cu/Pt micromotors [44] (reprinted with permission from the American Chemical Society).

Typical bubble-propelled CdS/C_60_ tubular micromotors with different catalysts (Pt, Pd, and MnO_2_) have been reported in the literature [67], as illustrated in Figure 6(a). It was found that the electrons in CdS quantum dots are trapped in the catalytic layer under light irradiation. The electrons react with oxygen and H_2_O_2_ is generated, which is then decomposed into O_2_ to propel the micro/nanomotors. Similarly, Zhang et al. developed a TiO_2_ bubble-driven micromotor coated with Au nanoparticles to promote the photocatalytic ability of micromotors through the surface plasmon effect [68], as shown in Figure 6(b).

For bubble-propelled micromotor systems, light can be used as a switch to control the motion of micro/nanomotors. During the switching process, the environment of micro/nanomotors can be controlled by light irradiation, which in turn affects the movement of the motors. A microbubble-driven Ti/Cr/Pt catalytic microengine in the H_2_O_2_ solution was reported [70], as shown in Figure 6(c). As is known, the concentrations of hydrogen peroxide and surfactant play important roles in the motion of bubble-driven micro/nanomotors. In this study, light could locally induce a decrease in the concentrations of the hydrogen peroxide fuel and surfactant to generate more giant bubbles, thus stopping the microengines. By decreasing the light intensity, the self-propulsion of microengines can be restarted. It was found that by adding spiropyrans to the bubble-propelled Cu/Pt micromotor, the motion of the Cu/Pt micromotor can be switched by UV and visible light [44], as shown in Figure 6(d). The photoisomerization of spiropyrans significantly affects the surface tension for bubble formation, thus affecting the movement of the micromotor. Under UV irradiation, spiropyrans are converted into corresponding isomers (e.g., merocyanine) with an increase in the surface tension; this influences the formation of bubbles and further decreases the speed of the micromotor. When the micromotor is exposed to visible light, the opposite effect is presented, i.e., the Cu/Pt micromotors start to move again.

### 3.4. Photochromic Reaction Propulsion

Taking advantage of the changes in the physical and chemical properties of photochromic materials during *cis-trans* isomerization, visible-light-driven rotary or linear molecular motors, interfacial tension gradient-driven micro/nanomotors, and liquid crystal elastomers can be designed.

**Figure 7:**
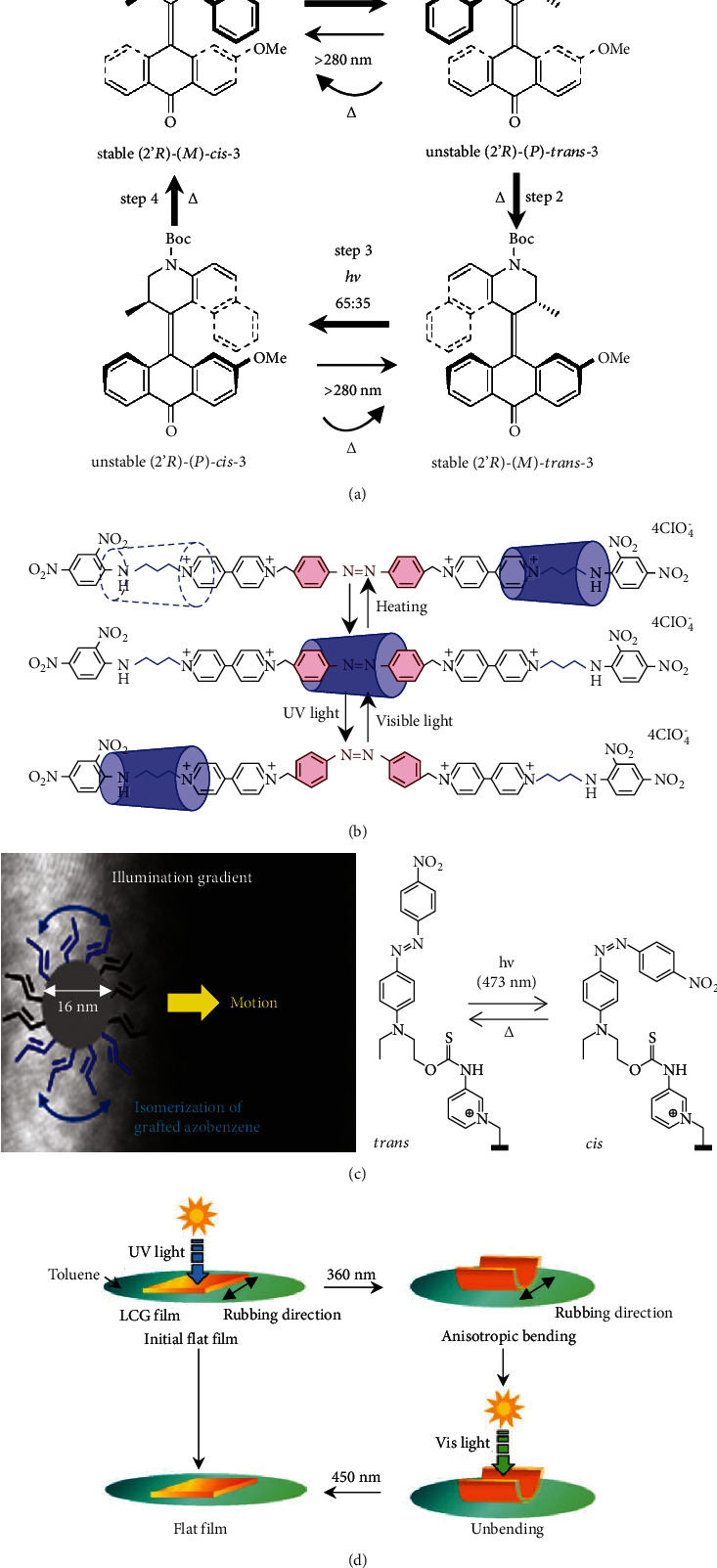
Propulsion mechanism of VLD-MNMs based on photochromic reactions. Rotary and linear molecular motors: (a) photochemical and thermal isomerization [107] (reprinted with permission from the American Chemical Society) and (b) schematic of the linear molecular motors [108] (reprinted with permission from the American Chemical Society). Interfacial tension gradient-driven micro/nanomotors: (c) azobenzene-coated polymer nanoparticles [30] (reprinted with permission from the American Chemical Society). Liquid crystal elastomers: (d) deforming microrobots [109] (reprinted with permission from John Wiley & Sons, Inc.).

1. *Visible-Light-Driven Rotary or Linear Molecular Motors*. Visible-light-driven rotary molecular motors are important manifestations of molecular motors, which can achieve unidirectional or continuous rotary motion. Due to the interaction between photochemical *cis-trans* isomerization and thermal isomerization, the molecular motors present rotary motion [45, 46, 107], as shown in Figure 7(a). Rotaxanes, as typical linear molecular motors, are compounds composed of an axle-type molecule surrounded by a macrocyclic component. In rotaxanes containing two different recognition sites in the axle component, the macrocyclic component can translate along the axle between these two “stations” by an external stimulus such as light and heating. The linear molecular motors based on photoisomerization reactions have been described in the literature [108], as shown in Figure 7(b).
2. *Interfacial Tension Gradient-Driven Micro/Nanomotors*. By utilizing the photoisomerization properties of photochromic materials, the interfacial properties, e.g., surface tension and wettability, of micro/nanomotors can be adjusted by surface coating. The local interfacial tension gradient resulting from the change in surface properties can generate a driving force and propel the micro/nanomotors. An excellent example of this was presented by Abid et al. that azobenzene-coated polymer nanoparticles with a diameter 16 nm can move with an illumination gradient [30], as shown in Figure 7(c).
3. *Liquid Crystal Elastomers*. Photochromic materials can be added to a liquid crystal polymer as a photoresponsive group. Under light irradiation, the liquid crystal elastomers produce relatively large deformations such as reversible contraction and expansion. Their contraction and expansion depend on the order-disorder phase transitions and order-order alignment changes caused by the isomerization of photochromic molecules, respectively. Taking advantage of the reversible contraction and expansion, the liquid-crystalline gels can perform the bending and unbending behavior in toluene [109], as shown in Figure 7(d). Similarly, due to the asymmetric contraction and expansion, the liquid crystal elastomer can move or form a capillary force inside it. Thus, a strategy to manipulate fluid slug by the photoinduced asymmetric deformation of tubular microactuators can be achieved [110]. Owing to the light intensity gradient, the tubular structure starts to deform and generates a capillary force to propel liquids. Additionally, sophisticated biomimetic motions, such as the peristalsis of annelids, can be achieved using photochromic liquid crystal elastomers [111].

As abovementioned, each propulsion mechanism has a unique advantage and an inherent defect. For instance, light-induced self-electrophoresis propulsion is dependent on the ion concentration of the solution, and bubble-driven VLD-MNMs can present fast motion with the addition of a surfactant. Considering this, the light-induced bubble-driven mechanism may exhibit unique advantages for application in wastewater treatment. Contrarily, as self-electrophoresis propulsion is sensitive to ion concentration, it may be used in the biomedical field for microsensing. Future studies on propulsion mechanisms will be focused on the achievement of VLD-MNMs with higher speed, greater driving force, and better adaptability. Hence, developing VLD-MNMs that can couple multiple propulsion modes may be the solution to satisfy the abovementioned requirements. For instance, developing microscale light-induced bubble-driven VLD-MNMs for long-distance motion and loading nanoscale self-electrophoresis VLD-MNMs on them for sensing will boost the advancement of VLD-MNMs. In summary, we expect that VLD-MNMs can operate in high-viscosity (plasma), high-ion concentration (sea water), and organic-water mixture (oil pollution) environments.

## 4. Controllable Methods

It is essential to control the movement of micro/nanomotors for their applications, especially in environmental remediation and cargo delivery. However, precise control remains a huge challenge because of the strong Brownian motion of particles at the micro/nanoscale. For VLD-MNMs, light as an external physical field to power the motor systems is easy to control. It is convenient to realize the motion behavior of micro/nanomotors by adjusting parameters such as the direction, intensity, wavelength, and polarization of external visible light. Controllable methods for VLD-MNMs mainly include speed control, directional control, and cluster control methods. Their controllable methods have been discussed hereinafter.

### 4.1. Speed Control

**Figure 8:**
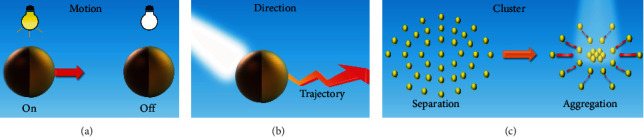
Controllable methods for VLD-MNMs. (a) The on/off motional control of VLD-MNMs. (b) The positive/negative phototaxis behavior of VLD-MNMs. (c) The collective behavior of VLD-MNMs.

Speed control of micro/nanomotors can be easily achieved by simply using light irradiation. At first, the on/off motional control is achieved by repeating the visible light on/off cycle, as illustrated in Figure 8(a). Several studies have been reported on the speed control of VLD-MNMs including inorganic and organic VLD-MNMs [29, 58, 65]. The fast/low motional behavior can be realized by changing the intensity and wavelength of visible light. Numerous results have shown that the motor speed increases with the increasing light intensity [51]. The different approach of combining a light field with an external physical field can also control the speed of micro/nanomotors. Liang et al. demonstrated that the semiconductor nanowires placed in an electric field can rapidly change their alignment direction and speed when irradiated by visible light [112].

### 4.2. Directional Control

**Figure 9:**
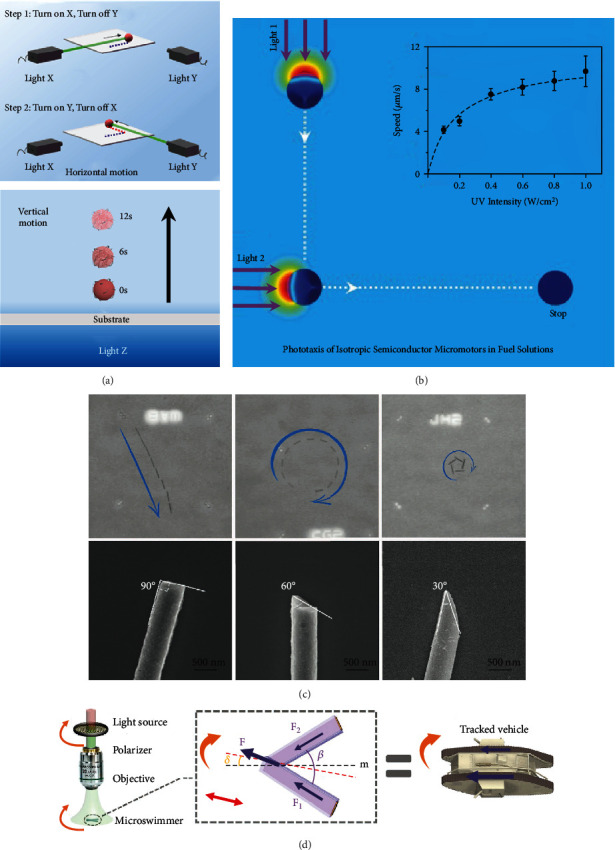
Directional control of VLD-MNMs. Incident light direction: (a) Cu_2_O@N-CNT micromotors [58] (reprinted with permission from the American Chemical Society) and (b) isotropic semiconductor micromotors [41] (reprinted with permission from John Wiley & Sons, Inc.). Shape of micro/nanomotors: (c) Si nanowires [53] (reprinted with permission from John Wiley & Sons, Inc.). Polarized light: (d) schematics of the polarotactic driving system based on differential steering similar to a tracked vehicle [56] (reprinted with permission from John Wiley & Sons, Inc.).

The direction of most of the micro/nanomotors can be adjusted by an external magnetic field [113–115]. However, light-driven micro/nanomotors present phototaxis or negative phototaxis behavior that can be used to achieve directional control by changing the direction of incident light without the external magnetic field, as illustrated in Figure 8(b). The phototaxis and negative phototaxis behaviors of micro/nanomotors are due to the asymmetric structure of the micro/nanomotors or the asymmetric photo exposure, which results in asymmetric chemical reactions. Based on this principle, Wang et al. developed a method to control the moving direction of micro/nanomotors by adjusting the incident light direction [58]. Under asymmetric visible-light irradiation, the side of the micromotor with light irradiation will generate a photocatalytic decomposition reaction of glucose, thus forming a molecular-concentration gradient around the micromotor surface. Nonelectrolyte diffusiophoresis drives the fluid flow to the side with a lower molecular concentration; this makes the micromotor exhibit the negative phototaxis behavior. Thus, the direction of the micromotor movement can be controlled by adjusting the direction of light irradiation, as shown in Figure 9(a). Regarding the phototaxis and negative phototaxis behaviors, similar approaches were presented [41], as shown in Figure 9(b).

In addition to the incident light direction, the shape of the micro/nanomotors can affect the direction of their movement. Wang et al. found a way to adjust the angular speed by altering the shape of nanomotors [53], as shown in Figure 9(c). These nanomotors presented different trajectories, such as “linear,” “circular,” and “rotational,” with different angles.

Compared with changing the characteristics of micro/nanomotors, adjusting the parameters of light irradiation is more convenient. Zhan et al. demonstrated a dichroic nanomotor based on Sb_2_Se_3_-ZnO core-shell nanowires, which can be activated with linear polarized light [56]. The thrust of the nanomotors can be regulated corresponding to the polarization angles, as illustrated in Figure 9(d).

### 4.3. Cluster Control

The inferior load capacity and short motion distance of individual micro/nanomotors limit their application in biomedical and wastewater treatment. However, the schooling behaviors of multiple micro/nanomotors, which can solve this problem, have become a hot research direction. Under visible-light irradiation, VLD-MNMs show different cluster forms, as shown in Figure 8(c).

**Figure 10:**
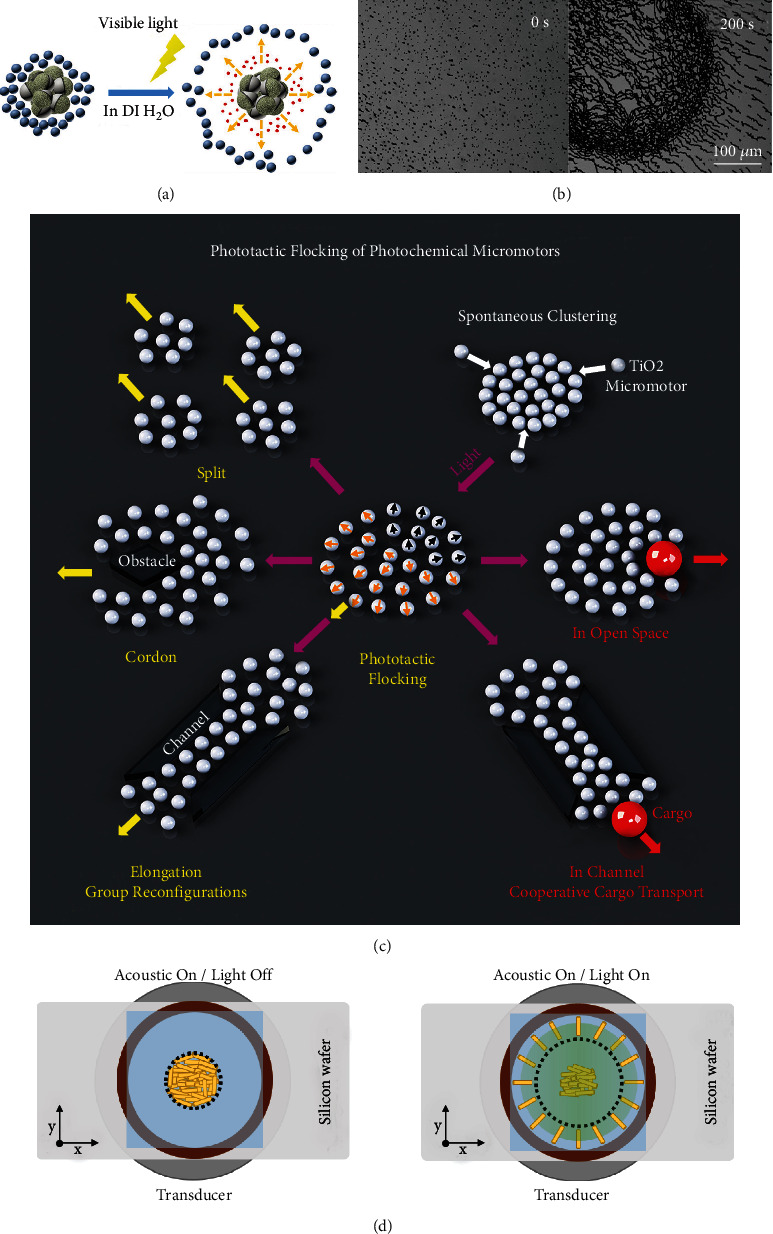
Collective behavior of VLD-MNMs. (a) PS/Ag/AgCl clusters [64] (reprinted with permission from John Wiley & Sons, Inc.). (b) Hematite peanut-shaped colloid motors [60] (reprinted with permission from John Wiley & Sons, Inc.). (c) TiO_2_ micromotor cluster [116] (reprinted with permission from Elsevier). (d) Au nanomotor clusters [117] (reprinted with permission from John Wiley & Sons, Inc.).

Individual micro/nanomotors can release different chemical molecules or ions under visible-light irradiation, which will interact with the surrounding colloidal particles or environment. For example, the collective behavior of an active Ag/AgCl micromotor and passive PS bead system was illustrated by Wang et al. [64], showing that the Ag/AgCl micromotors exclude the surrounding PS beads under visible-light irradiation, as shown in Figure 10(a). Similar collective behaviors of micro/nanomotors have been reported in the literature [60, 116], as shown in Figures 10(b) and 10(c), respectively.

A laser can also be used for the cluster control of micro/nanomotors. A sequential approach to achieve the collective behavior resulting from chemical gradients through diffusiophoretic interactions was introduced by Aubret et al. [42]. By borrowing energy from the hydrogen peroxide fuel, the hematite microswimmers assemble into self-spinning micro gears or rotors. However, the interactions disappear, and the cluster is destroyed by thermal turbulence upon turning off the light.

The combination of light and external acoustic sources or other methods is also popular for controlling the cluster behavior of micro/nanomotors. The collective behavior of Au nanomotors was controlled by the combination of light field and ultrasound field by Zhou et al. [117]. It was found that the nanomotors gathered at the pressure node in the acoustic field, showing a “firework” separation behavior under visible-light irradiation, as displayed in Figure 10(d). This collective behavior was also observed using other materials such as palladium, gold-coated silicon dioxide, and polypyrrole.

To date, several methods have been employed to control the collective behavior of VLD-MNMs, and significant efforts have been made in this regard. However, the existing control methods and strategies are relatively simple, i.e., most of the VLD-MNMs can only move or aggregate in two dimensions. Relying on the adjustment of the light field is not sufficient to satisfy further requirements. As abovementioned, controlling the VLD-MNMs by multiple physical field coupling will be beneficial for them to achieve a three-dimensional motion and aggregation. Meanwhile, the existing control strategies and equipment are oversimplified. We believe that a highly promising and useful method to control the collective behavior of VLD-MNMs is the employment of artificial intelligence (AI). We expect that the VLD-MNMs can respond to environmental variation, independently deliver or unload cargo, automatically plan trajectory, achieve complex three-dimensional motion and aggregation, etc. For example, when a VLD-MNM is added to wastewater, it can analyze the composition of the solvent, choose the cleaning method by itself, and automatically change its shape when it needs to pass through a slit. However, it is still a big challenge to combine VLD-MNMs with artificial intelligence.

## 5. Applications

Owing to their advantages, such as positive/negative phototaxis, easy control, and fast response to light irradiation, VLD-MNMs show a variety of potential applications such as in wastewater treatment, micropumps, cargo delivery, and sensing in micro/nanoscale. Moreover, visible light accounts for a higher proportion of sunlight than ultraviolet and near-infrared light, which makes VLD-MNMs show substantial potential in wastewater treatment than the other light-driven micro/nanomotors.

### 5.1. Applications in Wastewater Treatment

With the rapid growth of the global population and the improvement of industrialization, a large number of toxic effluents, including poisonous-organic solvents and heavy metal salts, are discharged into the ecosystem. Wastewater treatment is an urgent problem for humans. Compared with traditional methods, e.g., filtration, adsorption, and catalytic degradation, VLD-MNMs have unique advantages as they can autonomously move to accelerate the decomposition process [118].

**Figure 11:**
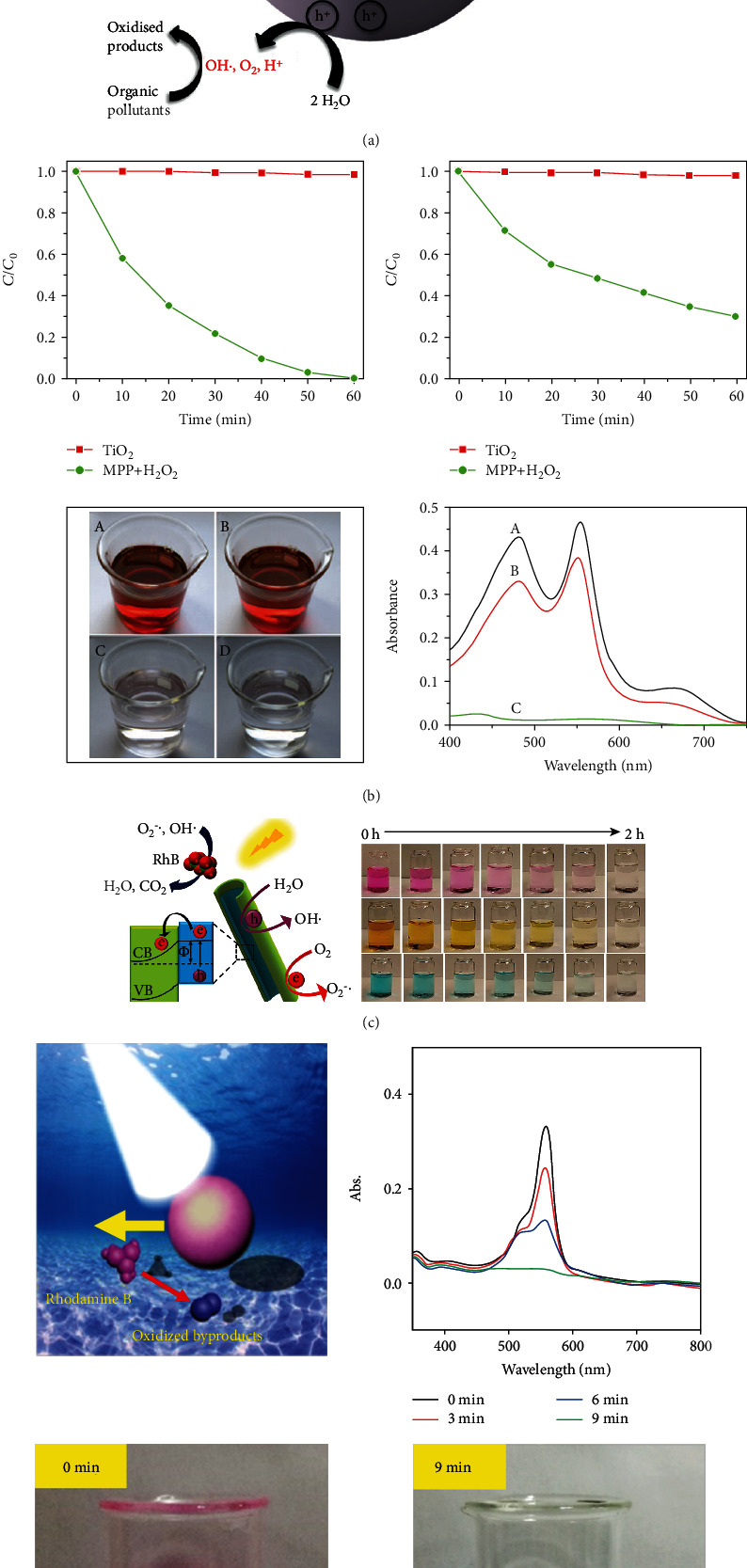
VLD-MNMs in wastewater treatment. (a) TiO_2_-Mo_7_-Au nanomotors [65] (reprinted with permission from the Royal Society of Chemistry). (b) Photocatalytic degradation of rhodamine B, methyl orange, and the organic mixture by a motor plasmonic photocatalyst with hydrogen peroxide under solar irradiation [68] (reprinted with permission from the Royal Society of Chemistry). (c) TiO_2_-PtPd nanotubes [54] (reprinted with permission from John Wiley & Sons, Inc.). (d) FePc micromotor [66] (reprinted with permission from the American Chemical Society).

Visible-light-driven titanium-based micromotors can photocatalytically degrade chemical and biological pollutants and hence can be widely used in wastewater treatment. Mallick and Roy reported a TiO_2_-Mo_7_-Au nanomotor that can be used to remove methylene blue and benzyl bromide from water [65], as shown in Figure 11(a). To more efficiently degrade the organic pollutants, Arabatzis et al. deposited gold nanoparticles onto titania thin films, which led to two times faster degradation of methyl orange than the sample without gold nanoparticle deposition [119]. Besides these studies, there are numerous other works focusing on the use of titanium-based micro/nanomotors for wastewater treatment [54, 68], as shown in Figures 11(b) and 11(c), respectively.

In addition to titanium-based micromotors, some other materials have significant potential in wastewater treatment. As illustrated in Figure 11(d), an iron phthalocyanine- (FePc-) and gelatin-based micromotor can move with water as a fuel under visible-light irradiation [66], which exhibits the self-diffusiophoresis mechanism. The FePc-based micromotor can be used to degrade organic pollutants, such as RhB, in the field of wastewater treatment.

### 5.2. Other Applications

**Figure 12:**
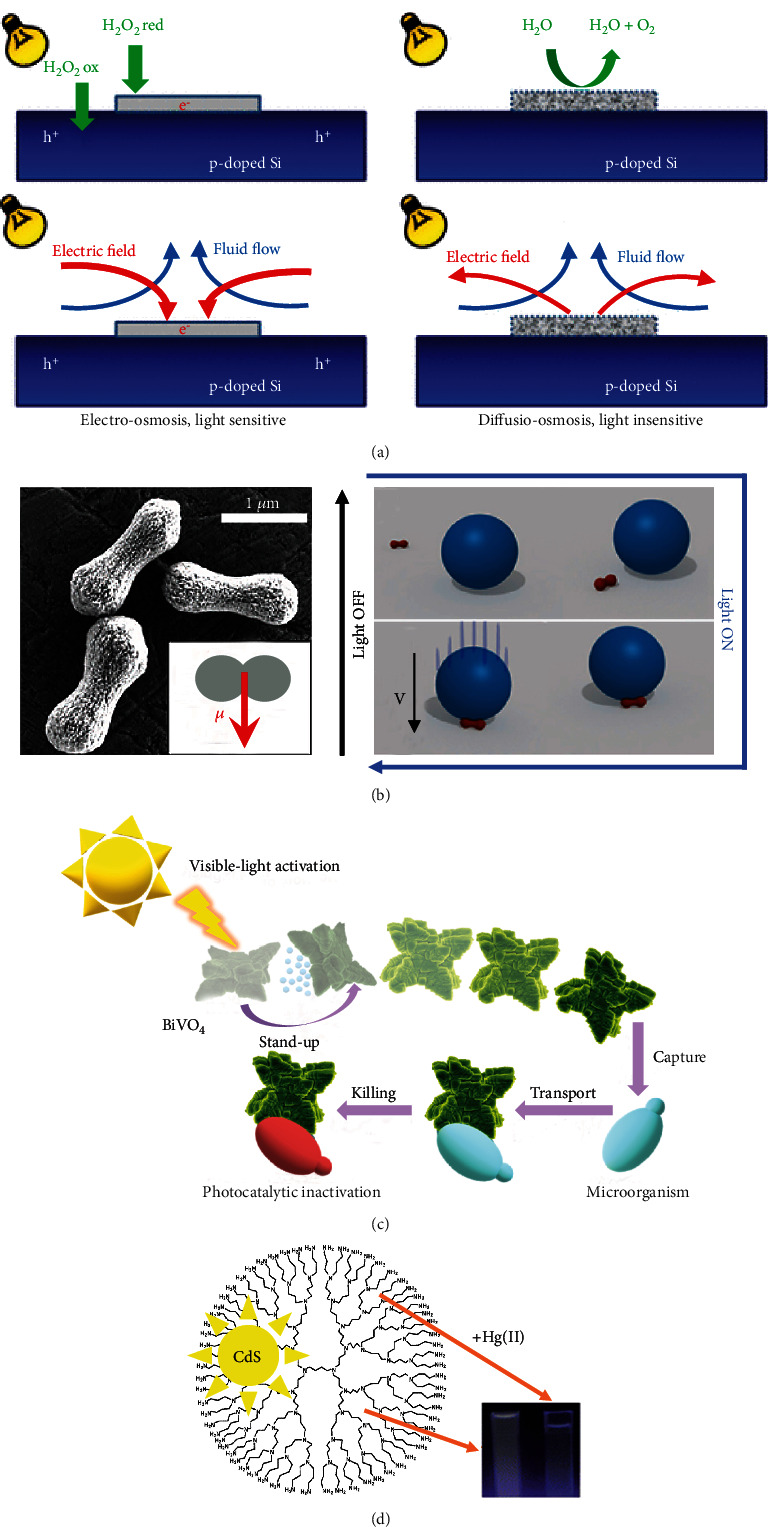
VLD-MNMs for micropumps, cargo delivery, and sensing. (a) Schematic of the visible-light-driven Si/Pt micropumps [120] (reprinted with permission from the American Chemical Society). (b) An SEM image of the hematite peanut particles and the schematic of docking [86] (reprinted with permission from the American Chemical Society). (c) Visible light photoactivated BiVO_4_ micromotors for controlled cargo transportation [43] (reprinted with permission from the American Chemical Society). (d) Schematic of the CdS–dendrimer nanocomposites for mercury sensing [121] (reprinted with permission from the Royal Society of Chemistry).

Micropumps, as typical micromachines, show excellent advantages for driving fluid flow. They can be used in drug transportation and microfluidics owing to their miniaturized overall size and high dosing accuracy. Considering this, Zhang et al. reported visible-light-driven semiconductor/metal (Si/Pt) micropumps that have two competing chemomechanical mechanisms [120], as shown in Figure 12(a). The light-sensitive electroosmotic and the light-insensitive diffusion-osmotic mechanisms have been illustrated in the literature, which depends on the catalyst roughness. In addition to the surface state of the micropumps, the interaction of positive and negative tracers with the micropumps is significantly different. Similar work was also reported by Esplandiu et al. [122]. Taking advantage of the speed control by visible light in the micropump system, they controlled the spatial distribution of colloidal microparticles in the liquid and patterned colloidal microparticle structures at specific locations on a wafer surface. An additional application of VLD-MNMs is in cargo transportation. Palacci et al. demonstrated a hematite micromotor that can dock a small particle, transfer it to the desired location, and freely release it [86], as shown in Figure 12(b). To reduce the external physical field or control complexity, Villa et al. developed star-shaped single-component BiVO_4_ micromotors [43], as shown in Figure 12(c). In addition, the fluorescence “on-off” strategy is one of the typical sensing approaches, which can be used in the sensing applications of VLD-MNMs [123]. Taking advantage of the unique optical properties, such as changes in photoluminescence, quantum dots can be used in the chemical sensing of ions. Based on the quenching of the fluorescence, Campos et al. reported the CdS quantum dots for mercury sensing [121]. The CdS quantum dots modified with polypropylenimine tetrahexacontaamine dendrimer generation 5 for the incorporation of fluorescence CdS, as shown in Figure 12(d). Inspired by this strategy, Pacheco et al. reported a visible-light-driven CdTe-Fe_3_O_4_ Janus micromotor, which can be used for heavy metal removal and sensing [80]. The photoresponsive CdTe quantum dots can interact with Hg^2+^, resulting in the cation exchange of Cd^2+^ to HgTe. The Hg^2+^ concentration can be monitored through the fluorescence decay of the micromotors.

To date, water treatment and employment in the biomedical field are two key directions for the further applications of VLD-MNMs. However, most of the demonstrations are still in the laboratory with several limitations restricted by cost, efficiency, and scale. In water treatment, the expansion of VLD-MNMs from laboratory demonstration to industrial application, i.e., from microscale to macroscale, is still proceeding. We expect that billions of VLD-MNMs will be distributed into the ocean by plane and used to treat the marine oil pollution. In biomedical engineering, we believe that VLD-MNMs will have significant potential in eye disease treatment because of their harmlessness. In the coming years, VLD-MNMs may be injected into the eyeball and can be used to conduct surgery for the treatment of eye diseases, such as glaucoma, upon their arrival at the affected area.

## 6. Conclusion

Considerable attention has been paid to VLD-MNMs as they are driven by visible light that accounts for a large proportion of solar energy and is necessary for living organisms; VLD-MNMs can be widely used in the field of wastewater treatment, cargo transportation, micropumps, sensing, etc. In this review, we presented an overview of the recent progress in the field of VLD-MNMs starting from the fundamentals to their applications: photoactive materials, propulsion mechanisms, controllable methods, and potential applications. Considering the limits of the visible-light wavelength range, photocatalytic and photosensitive materials are generally used for most of the inorganic micro/nanomotors. In contrast, photochromic materials are used for organic micro/nanomotors. Self-electrophoresis, self-diffusiophoresis, bubble propulsion, and photochromic reaction propulsion are the main propulsion mechanisms of VLD-MNMs. It was found that the speed, directional, and cluster control of VLD-MNMs can be achieved by adjusting the light irradiation parameters or by introducing external physical fields.

For the photoactive materials used in VLD-MNMs, the existing photocatalytic materials have weak photocatalytic efficiency and visible-light absorption performance, which can be improved through changing the crystal structure, the surface state, and the morphology of the materials. Besides these, doping is also a promising strategy. Furthermore, the combination of the organic photoactive materials and the inorganic photoactive materials is also a promising strategy to design new materials for VLD-MNMs, which may expand the application of VLD-MNMs with the advantages of both inorganic and organic materials.

In terms of the propulsion mechanisms, the performance for the motion of VLD-MNMs is still inferior, with slow speed, weak driving force, and poor adaptability. These are the main challenges for the VLD-MNMs to overcome in driving. As is well known, the bubble-driven VLD-MNMs present a higher motion behavior, which can be used in the improvement of the VLD-MNM motion capabilities. The self-electrophoresis propulsion mechanism is more sensitive to the ion concentration, which can be used for sensing. Therefore, the development of the VLD-MNMs that can couple multiple propulsion modes may be the solution to satisfy the efficient motion requirements.

Furthermore, the existing control methods are relatively simple and the cluster control of VLD-MNMs is inflexible, which is not sufficient to satisfy the application requirements. For more intelligent movement behavior and more effective control method, combining the design of VLD-MNMs with artificial intelligence is a promising method to enhance the response of VLD-MNMs to environmental variation and automatic control. Moreover, it would be very interesting to investigate the interaction, communication, and collective behavior of a large number of individual VLD-MNMs.

Although significant efforts have been made over the past few years, the research on VLD-MNMs is still at its infancy. However, we believe that VLD-MNMs will play an important role in the development of materials science, robotics, and nanotechnology owing to their excellent properties.

## Figures and Tables

**Table 1:** Representative VLD-MNMs and their corresponding shapes, propulsion mechanisms, and speeds.

Materials	Shape	Propulsion mechanism	Motion velocity (*μ*m s^−1^)	Light
Au/B-TiO_2_ [51]	Janus sphere	Self-electrophoresis	30.1	UV-Vis
Au/TiO_2_ [52]	Nanocap	Self-electrophoresis	—	Vis
n^+^-Si/p-Si [53]	Core-shell nanowire	Self-electrophoresis	9.6	Vis-NIR
Au/Fe_2_O_3_ [39]	Nanorod	Self-electrophoresis	30	Vis
TiO_2_/Si [40]	Nanotree	Self-electrophoresis	5	UV-Vis
TiO_2_/Pt/Pd [54]	Tubular	Self-electrophoresis	—	UV-Vis
Zn_x_Cd_1-x_Se/Cu_2_Se/Pt [55]	Core-shell nanowire	Self-electrophoresis	12	Vis
Au/Cu_2_O [38]	Janus sphere	Self-electrophoresis	6	Vis
Si/Au [37]	Nanotadpole	Self-electrophoresis	5	Vis
Au/BiOI [29]	Janus sphere	Self-electrophoresis	1.62	Vis
Sb_2_Se_3_/ZnO [56]	Core-shell nanowire	Self-electrophoresis	15	Polar
Pt/Ag [57]	Nanorod	Self-electrophoresis	9.21	UV-Vis
Cu_2_O/N-carbon [58]	Sphere	Self-diffusiophoresis	18.71	Vis
Pt/g-C_3_N_4_ [59]	Sphere	Self-diffusiophoresis	14	UV-Vis
Hematite colloid [60]	Peanut	Self-diffusiophoresis	1.84	Vis
Cu_2_O/TiO_2_/SiO_2_ [61]	Chevron	Self-diffusiophoresis	15	UV-Vis
Cu/TiO_2_ [62]	Janus sphere	Self-diffusiophoresis	43	UV-Vis
PMMA/AgCl [63]	Janus sphere	Self-diffusiophoresis	12	UV-Vis
Ag/AgCl [64]	Janus sphere	Self-diffusiophoresis	70	Vis
TiO_2_-Mo_7_-Au [65]	Sphere	Self-diffusiophoresis	10.22	Vis
FePc [66]	Sphere	Self-diffusiophoresis	32	Vis
BiVO_4_ [43]	Microstar	Self-diffusiophoresis	5	Vis
Cu_2+1_O [36]	Sphere	Self-diffusiophoresis	107.32	Vis
CdS/C_60_/Pd (Pt, MnO_2_) [67]	Tubular	Bubble propulsion	1058	UV-Vis
PDA/Pt/TiO_2_/Au [68]	Core-shell sphere	Bubble propulsion	130	Solar
ZnO/ZnO_2_/Pt [69]	Janus sphere	Bubble propulsion	350	UV-Vis
Spiropyrans/Cu/Pt [44]	Tubular	Bubble propulsion	57	UV-Vis
Ti/Cr/Pt [70]	Tubular	Bubble propulsion	65	Vis
Azobenzene-coated [30]	Sphere	Interfacial tension gradient	16.9	UV-Vis
Alkenes [46]	Molecular rotary motor	Photochemical reaction	—	UV-Vis
Rotaxane [47]	Molecular shuttle	Photochemical reaction	—	UV-Vis
[2]Rotaxane [50]	Molecular switch	Photochemical reaction	—	UV-Vis

**Table 2:** Classification of conventional photoactive materials.

Photoactive materials	Inorganic photoactive materials	Photocatalytic materials	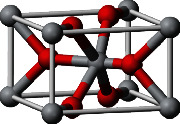
		Photosensitive materials	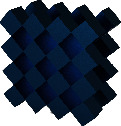
		Photothermal materials	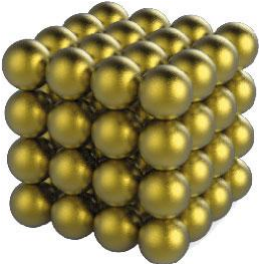
	Organic photoactive materials	Photochromic materials	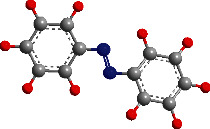
		Dyes	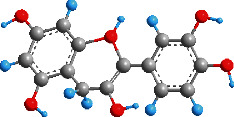

**Table 3:** Materials used in VLD-MNMs and their energy bands [75].

Material	*E*_*g*_ (eV)	CB vs. NHE (eV)	VB vs. NHE (eV)
In_2_O_3_ [76]	2.8	-0.62	2.18
C_3_N_4_ [77]	2.7	-1.3	1.4
BiVO_4_ [43]	2.5	0.29	2.79
CdS [67]	2.4	-0.52	1.88
Cu_2_O [38]	2.2	-0.28	1.92
Fe_2_O_3_ [39, 78]	2.2	0.28	2.48
BiOI [29]	1.86	0.01	1.87
B-TiO_2_ [51]	1.75	—	—
CdSe [79]	1.7	-0.6	1.1
CdTe [80]	1.4	-1.0	0.4
Si [53]	1.1	-0.5	0.6
MnO_2_ [67]	0.25	1.33	1.58
Fe_3_O_4_ [80]	0.1	1.23	1.33
